# Radiomics models for diagnosing microvascular invasion in hepatocellular carcinoma: which model is the best model?

**DOI:** 10.1186/s40644-019-0249-x

**Published:** 2019-08-28

**Authors:** Ming Ni, Xiaoming Zhou, Qian Lv, Zhiming Li, Yuanxiang Gao, Yongqi Tan, Jihua Liu, Fang Liu, Haiyang Yu, Linlin Jiao, Gang Wang

**Affiliations:** 1 Department of Radiology, The Affiliated Hospital of QingDao University, QingDao, ShanDong China; 2 College of Computer Science and Engineering, Shandong University of Science and Technology, Qingdao, Shandong China; 3 Intervention Medical Center, The Affiliated Hospital of QingDao University, QingDao, ShanDong China

**Keywords:** Hepatocellular carcinoma, Microvessel, Neoplasm invasiveness, Radiomics

## Abstract

**Objectives:**

To explore the feasibility of diagnosing microvascular invasion (MVI) with radiomics, to compare the diagnostic performance of different models established by each method, and to determine the best diagnostic model based on radiomics.

**Methods:**

A retrospective analysis was conducted with 206 cases of hepatocellular carcinoma (HCC) confirmed through surgery and pathology in our hospital from June 2015 to September 2018. Among the samples, 88 were MVI-positive, and 118 were MVI-negative. The radiomics analysis process included tumor segmentation, feature extraction, data preprocessing, dimensionality reduction, modeling and model evaluation.

**Results:**

A total of 1044 sets of texture feature parameters were extracted, and 21 methods were used for the radiomics analysis. All research methods could be used to diagnose MVI. Of all the methods, the LASSO+GBDT method had the highest accuracy, the LASSO+RF method had the highest sensitivity, the LASSO+BPNet method had the highest specificity, and the LASSO+GBDT method had the highest AUC. Through Z-tests of the AUCs, LASSO+GBDT, LASSO+K-NN, LASSO+RF, PCA + DT, and PCA + RF had Z-values greater than 1.96 (*p*<0.05). The DCA results showed that the LASSO + GBDT method was better than the other methods when the threshold probability was greater than 0.22.

**Conclusions:**

Radiomics can be used for the preoperative, noninvasive diagnosis of MVI, but different dimensionality reduction and modeling methods will affect the diagnostic performance of the final model. The model established with the LASSO+GBDT method had the optimal diagnostic performance and the greatest diagnostic value for MVI.

## Keypoints

1. Radiomics can be used for the preoperative diagnosis of MVI in HCC patients.
2. The diagnostic performance of the models obtained by different dimension reduction methods and modeling methods is different.
3. The LASSO+GBDT method had the optimal diagnostic performance and the greatest diagnostic value for MVI.

## Introduction

Hepatocellular carcinoma (HCC) is one of the most common malignant tumors in the world. Of all malignant tumors, HCC has the sixth highest incidence and the third highest mortality [1–4]. The number of HCC patients in China accounts for 55% of the total number of HCC patients worldwide, and the mortality rate of HCC is increasing worldwide [5]. Currently, curative treatment for HCC is surgical resection, and liver transplantation [6, 7]. Unfortunately, postoperative recurrence remains an important problem for patients after HCC treatment. It has been reported that the recurrence rate of HCC is as high as 70% within 5 years after surgery and 30% within 5 years after liver transplantation [8]. Because of the high recurrence rate of HCC, the survival time of HCC patients is significantly shortened [9].

Vascular invasion, including both macrovascular and microvascular invasion (MVI), refers to invasive manifestation of tumors and is an independent risk factor for tumor recurrence after surgery [6, 10]. Macrovascular invasion is defined as invasion of the tumor into a major vessel, which can be identified during macroscopic examinations or radiological imaging; in contrast, MVI is defined as the presence of tumor cells in the endothelial-lined vascular lumen, which is only visible under a microscope [4].

Because MVI is histopathologically diagnosed, it is difficult to diagnose MVI by CT or MRI before surgery. However, early detection and treatment of HCC can reduce the recurrence rate, but HCC is often found in the middle and late stages [11–13]. Therefore, it is very important to determine whether MVI exists before surgery to help create further treatment plans and provide early intervention measures, which ultimately helps to reduce the recurrence rate for patients after hepatectomy or liver transplantation. At present, some studies have attempted to diagnose MVI preoperatively by radiologic imaging and specific laboratory tests, and some radiologic studies have shown that MVI is closely related to many factors, including tumor size, tumor number, tumor margin, histologic grade, gross classification of the HCC, among others [10, 14]. However, the feasibility of using these features to identify MVI is still controversial and depends on the subjective judgment of the diagnostic radiologists.

At present, diagnosis through imaging mainly relies on direct observation by radiologists. Radiologists use contrast differences between different tissues to identify diseases and analyze the factors for a diagnosis. The knowledge base, diagnostic experience, work status, etc. of the radiologist can, therefore, affect the accuracy of the diagnosis. Additionally, radiological images contain more information than what is visible to the clinician’s eye, and these “hidden” data can provide much more insight into the tissue of interest than previously thought. Thus, radiomics was born as a “new” method. Radiomics is defined as the high-throughput extraction of quantitative imaging features or textures (radiomics) to decode tissue pathology and create a high-dimensional data set for feature extraction [15, 16]. Radiomics aims to quantify tumor heterogeneity related to changes in cellularity, necrosis, angiogenesis, and extracellular matrix deposition in the tumor microenvironment.

Radiomics provides possibility for early and accurate diagnosis of MVI in HCC patients. Previous studies have shown that radiomics can identify MVI in patients with HCC preoperatively [17–19]. However, due to a lack of standardization in radiomics, there have been no studies that analyzed whether there are differences in the results obtained using different radiomics dimensionality reduction methods and modeling methods, and there are no research reports on which dimensionality reduction and modeling methods are most suitable for radiomics.

In this paper, we discuss the application of radiomics for diagnosing MVI by combining the dimensionality reduction and modeling methods commonly used in radiomics research, comparing different methods to evaluate the diagnostic performance of each model and determining the best radiomics model.

## Materials and methods

### Patients

The institutional review board of our hospital ethics committee approved this retrospective study, and the requirement for informed consent was waived. Patients were identified by searching the electronic HIS (hospital information system) database at our hospital between June 2015 and September 2018. The demographic, clinical and pathologic data were collected from electronic medical records.

The study inclusion criteria were as follows: (1) patients who underwent surgical resection or liver transplantation and had a histopathological diagnosis of HCC, (2) patients who received a dynamic contrast-enhanced CT examination of their upper abdomen in our hospital within 1 month before surgery, (3) patients with a diagnosis of HCC made on the basis of dynamic CT imaging data of a solitary hepatic tumor that showed hypervascular enhancement in the arterial phase and contrast washout in the equilibrium phase, (4) patients with no other hepatic inflammatory lesions, tumors or previous liver surgery history, and (5) patients with scans of excellent image quality.

The exclusion criteria were patients with (1) preoperative treatment, such as transcatheter arterial chemoembolization (TACE), radiofrequency ablation or partial hepatectomy, (2) lesions less than 10 mm in diameter, (3) imaging or pathological manifestations of macrovascular invasion, or (4) unclear lesion boundaries and ROIs that could not be outlined.

### Research methods

All CT examinations were performed with our institutional spiral CT scanner, which consisted of five different devices, including a 16-slice CT (GE Medical Systems, Milwaukee, WI) and a CT scanner with the newest dual-source detector CT (dual source and dual detector, 128 layers, Siemens Medical Systems, Erlangen, Germany). To ensure image consistency, the different devices used the same scanning parameters. The scanning parameters were 120 kV, 250–300 mAs, and a 512 × 512 matrix, 1 mm thickness and interval of the original image layer, and reconstruction to a level of 5 mm. After administering 90 ml of iodine contrast agent (350 mg I/ml) at a flow rate of 3.0 ml/s with a contrast injector, the dynamic contrast-enhanced acquisition was performed at fixed time points. The acquisition time periods were 30 s - 33 s for the arterial phase, 67 s - 70 s for the portal venous phase and 177 s–180 s for the equilibrium phase (adjustment for different devices). The raw data were acquired, and 5-mm axial images in the portal venous phase were selected for texture analysis. Imaging data were retrieved from the Picture Archiving and Communication System (Centricity PACS Radiology RA1000 Workstation, General Electric, Milwaukee, WI, USA) at our institution.

### Statistical analysis

SPSS 18.0.0 (Chicago, IL, USA) software was used for statistical analysis. Categorical variables are represented as numbers or percentages. Continuous variables are expressed as the mean ± standard deviation, and the comparisons among the categorical data were performed by the chi-square tests. A *p* < 0.05 was considered statistically significant. Dimensionality reduction and modeling methods were examined using PyCharm (developed by JetBrains.r.o., version 4.5.4, a free and open-source software based on the Python language). MATLAB (The MathWorks, Inc.) was used to compare area under curve (AUC) with Z-tests, and to conduct decision curve analysis (DCA).

## Workflow

### Feature extraction and selection

**Fig. 1:**
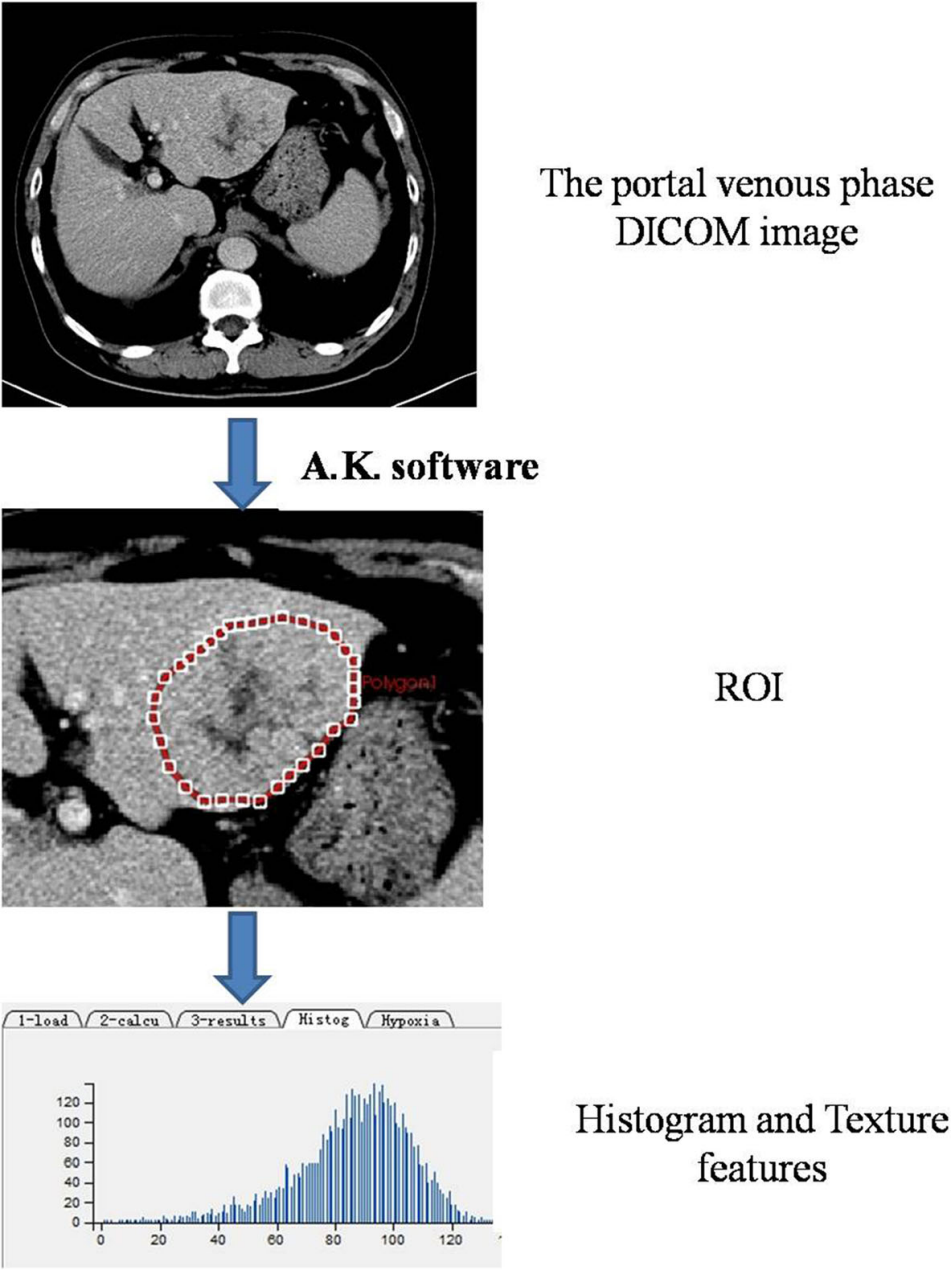
The steps of drawing an ROI. The 5 mm portal vein phase DICOM images of the largest cross-sectional area were exported to A.K. software The ROI was manually draw along the edge of the lesion

The 5-mm portal venous phase DICOM images of the largest cross-sectional area were exported to the A.K. software (GE Healthcare, China) for texture extraction. The images were assessed by a consensus reading from two radiologists with 12 years or 15 years of experience in abdominal imaging, and polygonal regions of interest (ROIs) were drawn around the margins of the tumor to separate the tumor from the surrounding normal liver parenchyma. The ROI size was as close as possible to the edge of the lesion but did not extend beyond the edge of the lesion (reducing the partial volume effect). The texture parameters were automatically obtained by the software, as is shown in Fig. 1.

### Data preprocessing

**Fig. 2:**
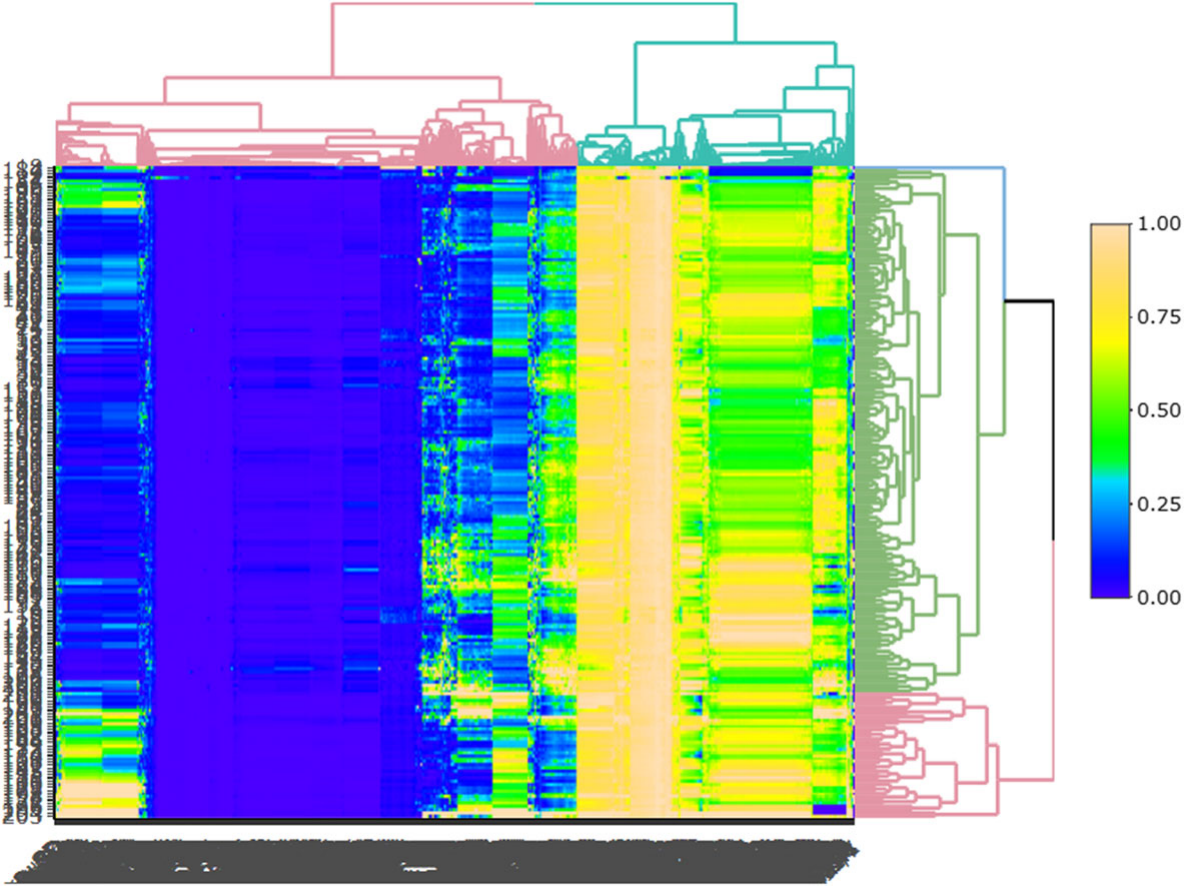
Radiomics heatmaps (normalized data). Heatmap depicting correlation coefficient matrix of 1044 features. Unsupervised clustering analysis was used. The heatmap represents the correlation between parameters. The stronger the correlation is, the larger the value (the lighter the color) is, and the worse the correlation is, the smaller the value (the darker the color) is

From each patient, 1044 sets of texture parameters were extracted using mean substitution to supplement some texture feature parameters with missing individual values. In this study, the ratio of MVI-positive to MVI-negative tumors was 118:88. We used a classic oversampling method, the SMOTE method, to process the data so that the ratio of the data in the two groups reached 1:1. Finally, we normalized the data so that all texture feature parameters were between (0, 1). The heatmap of normalized data is shown in Fig. 2.

### Dimension reduction and model construction

We used the three most common methods in radiomics research to reduce the dimensions of the preprocessed data: least absolute shrinkage and selection operator (LASSO), neighborhood rough set (NRS), and principal components analysis (PCA). After the dimension reduction, the data obtained from each dimension reduction were modeled by the following seven different modeling methods: back-propagation neural network (BPNet), k-nearest neighbors (KNN), support vector machine (SVM), random forest (RF), decision tree (DT), Bayes, and gradient boosting decision tree (GBDT). By combining different dimension reduction methods and modeling methods, 21 methods were applied for radiomics research in our study. We divided the data into a training set and verification set. There were 148 patients in the training set and 58 patients in the verification set. Moreover, the training set was subdivided into four training sets (118 patients) and one test set (30 patients) with a 5-fold cross-validation method. These four training sets were put into the model for training, and then the test sets were used to verify the performance of the model. The parameters were constantly adjusted to improve the model to obtain the best model. When an optimal model was obtained, the validation sets were used to test the diagnostic performance of the model.

### Model evaluation

A receiver operating characteristic (ROC) curve was used to assess the diagnostic performance of different models. The accuracy, sensitivity and specificity were obtained, and PyCharm software was used to draw the ROC curve and calculate the AUC. Z-tests for the AUC were obtained for the 21 methods using MATLAB and DCA concurrently.

## Results

A total of 206 patients with HCC were enrolled in this study. There were 88 patients in the MVI-positive group, including 74 males and 12 females, with an average age of 57 ± 1.2 years. There were 118 patients in the MVI-negative group, including 87 males and 26 females, with an average age of 59 ± 0.8 years. The statistical analysis showed that there were no significant differences in sex (χ2 = 3.192, *p* > 0.05) or age (χ2 = 39.633, *p* > 0.05) between the two groups.

**Fig. 3:**
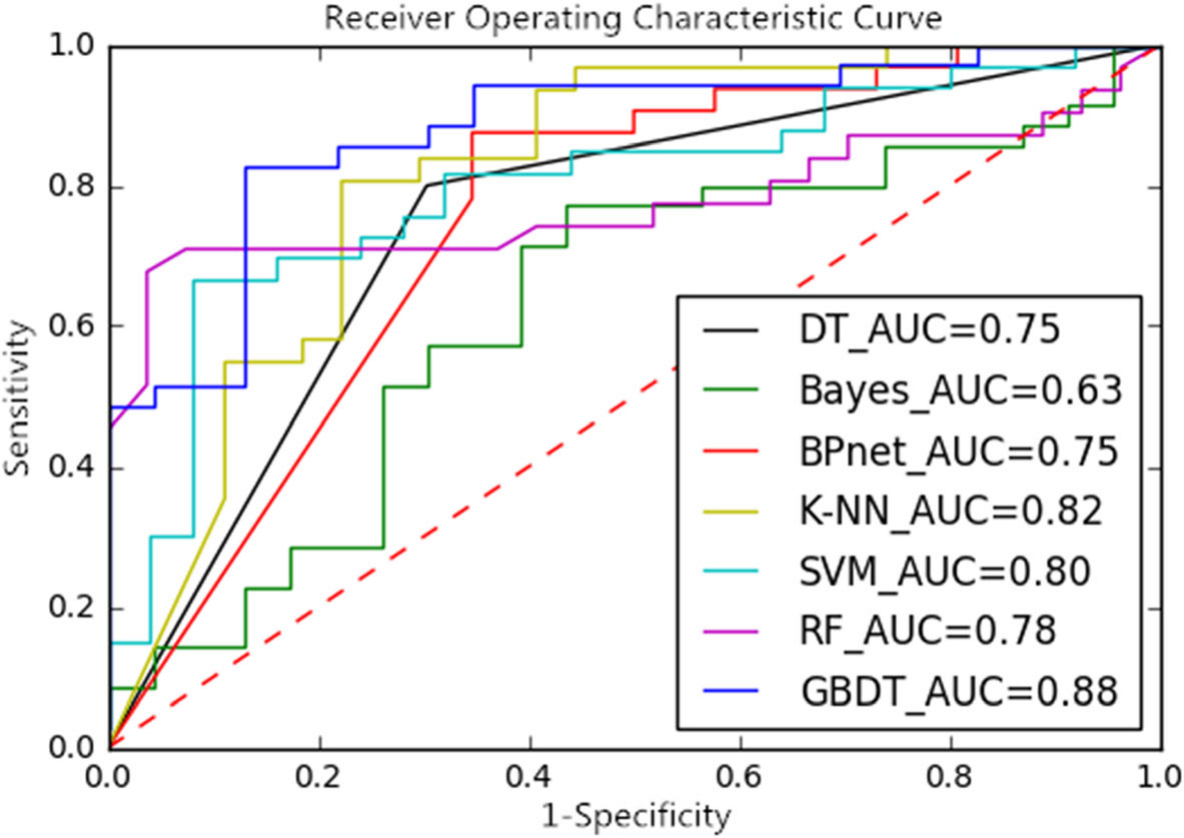
ROC Curves and AUCs of the Dimension-Reduced LASSO Model

**Fig. 4:**
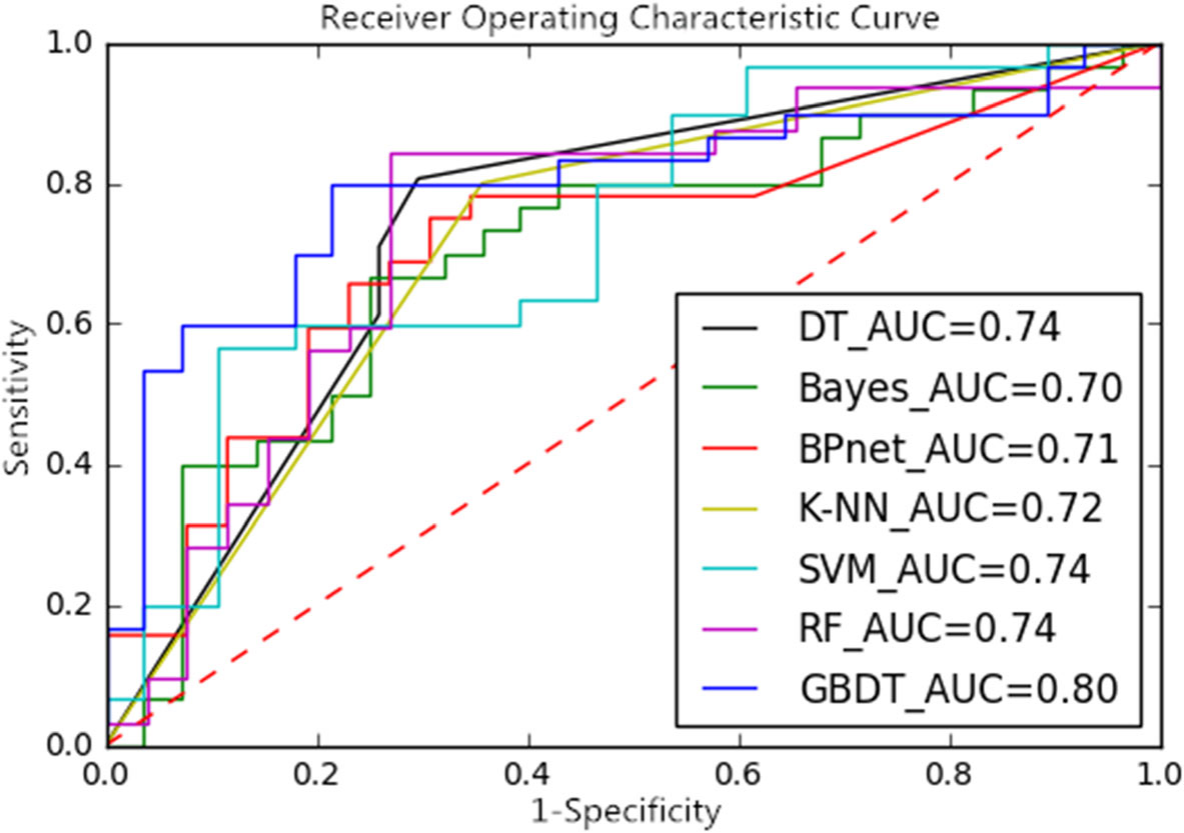
ROC Curves and AUCs of the Dimension-Reduced NRS Model

**Fig. 5:**
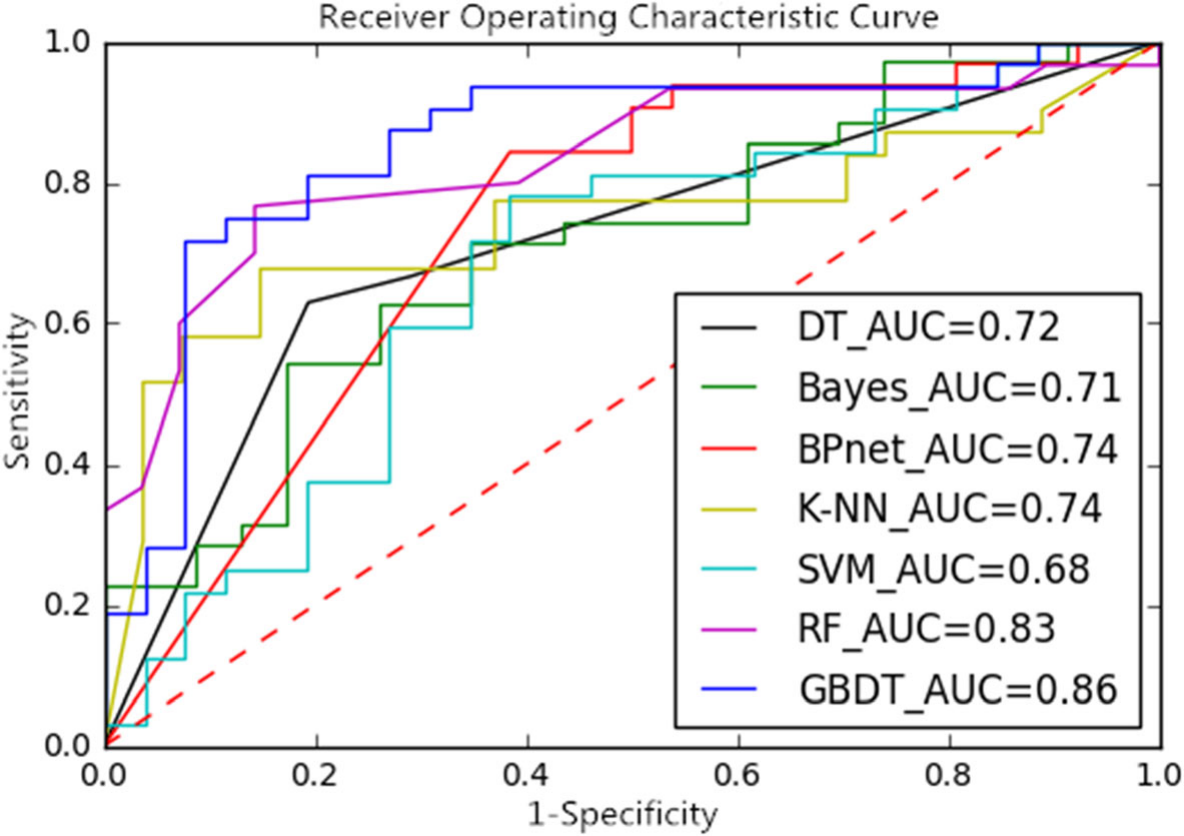
ROC Curves and AUCs of the Dimension-Reduced PCA Model

**Fig. 6:**
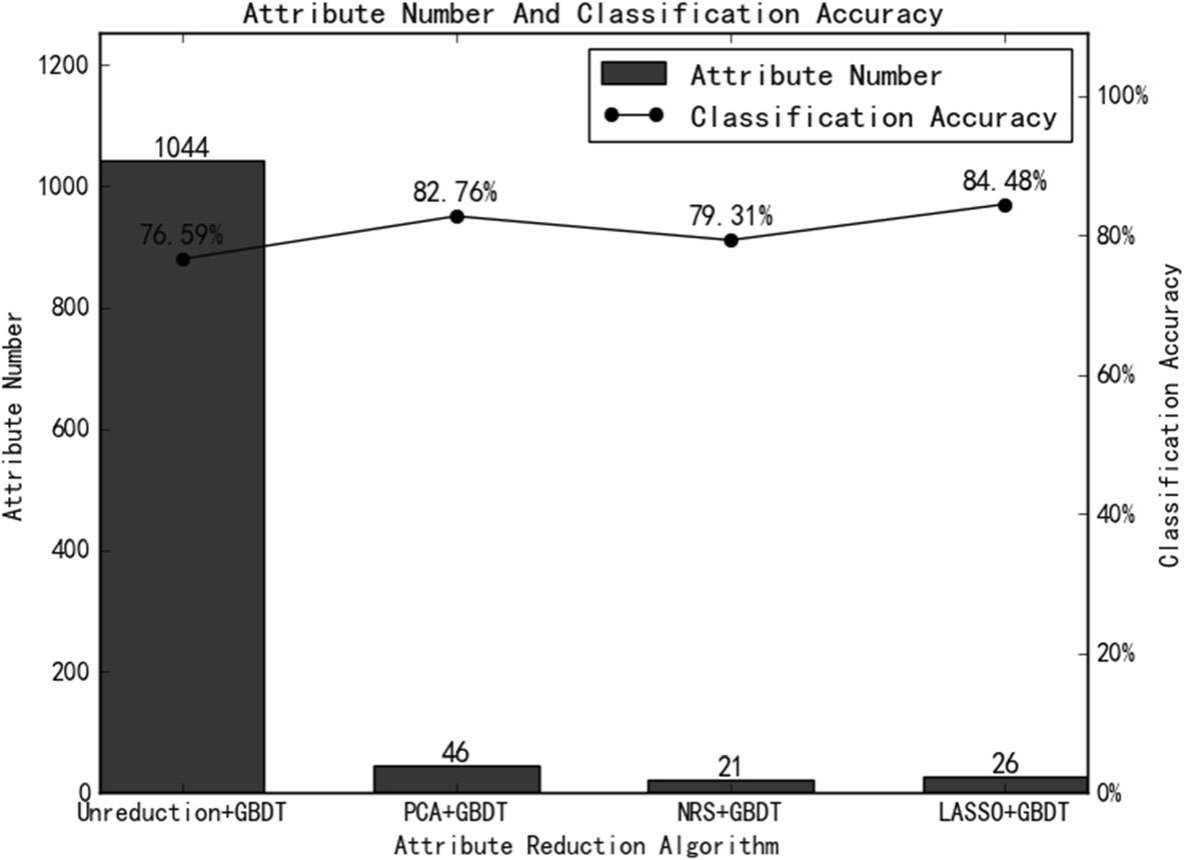
The Number of Attribute Reductions From the Different Dimension Reduction Methods and the Accuracy of the GBDT Model

Through different dimension reduction methods and combinations of various modeling methods, we finally obtained 21 methods for this study. Table 1, Table 2 and Table 3 show the accuracy, sensitivity and specificity of the final model obtained by combining the three-dimension reduction methods of LASSO, NRS and PCA with the seven modeling methods. The tables show that the LASSO+GBDT method had the highest accuracy (84.48%), the LASSO+RF method had the highest sensitivity (92.59%), and the LASSO+BPNet method had the highest specificity (87.50%). Figures 3, 4 and 5 are ROC curves of the diagnostic performance of the final model obtained by testing the 21 methods mentioned above. As the results show, all methods could be used to diagnosis MVI before surgical HCC treatment, but the diagnostic performance of the different methods was different. The AUCs of the 21 methods ranged from 0.63 to 0.88, and among these methods, the LASSO+GBDT method had the highest AUC. The results of the attribute reduction through different dimensionality reduction methods and the accuracy of the GBDT algorithm model are shown in Fig. 6.

**Table 1:** Results of each algorithm model after LASSO dimensionality reduction

	TP	FN	FP	TN	Accuracy	Sensitivity	Specificity
LASSO+DT	20	6	8	24	75.86%	76.92%	75.00%
LASSO+Bayes	13	10	8	27	68.97%	56.52%	77.14%
LASSO+BPnet	17	9	4	28	77.59%	65.38%	87.50%
LASSO+K-NN	23	7	4	24	81.03%	76.67%	85.71%
LASSO+SVM	23	2	11	22	77.59%	92.00%	66.67%
LASSO+RF	25	2	9	22	81.03%	92.59%	70.97%
LASSO+GBDT	19	4	5	30	84.48%	82.61%	85.71%

*FN False Negative, *FP* False Positive, *TN* True Negative, *TP* True Positive

**Table 2:** Results of each algorithm model after NRS dimensionality reduction

	TP	FN	FP	TN	Accuracy	Sensitivity	Specificity
NRS + DT	21	6	11	20	70.69%	77.78%	64.52%
NRS + Bayes	17	11	7	23	68.97%	60.71%	76.67%
NRS + BPnet	21	5	11	21	72.41%	80.77%	65.63%
NRS + K-NN	18	10	6	24	72.41%	64.29%	80.00%
NRS + SVM	23	5	6	24	70.69%	82.14%	80.00%
NRS + RF	19	7	5	27	79.31%	73.08%	84.38%
NRS + GBDT	23	5	7	23	79.31%	82.14%	76.67%

*FN False Negative, *FP* False Positive, *TN* True Negative, *TP* True Positive

**Table 3:** Results of each algorithm model after PCA dimensionality reduction

	TP	FN	FP	TN	Accuracy	Sensitivity	Specificity
PCA + DT	24	7	9	18	72.41%	77.42%	66.67%
PCA + Bayes	12	11	9	26	65.52%	52.17%	74.29%
PCA + BPnet	16	10	5	27	74.14%	61.54%	84.38%
PCA + K-NN	17	10	7	24	70.69%	62.96%	77.42%
PCA + SVM	16	10	9	23	67.24%	61.54%	71.88%
PCA + RF	24	4	7	23	81.03%	85.71%	76.67%
PCA + GBDT	21	5	5	27	82.76%	80.77%	84.38%

*FN False Negative, *FP* False Positive, *TN* True Negative, *TP* True Positive

**Fig. 7:**
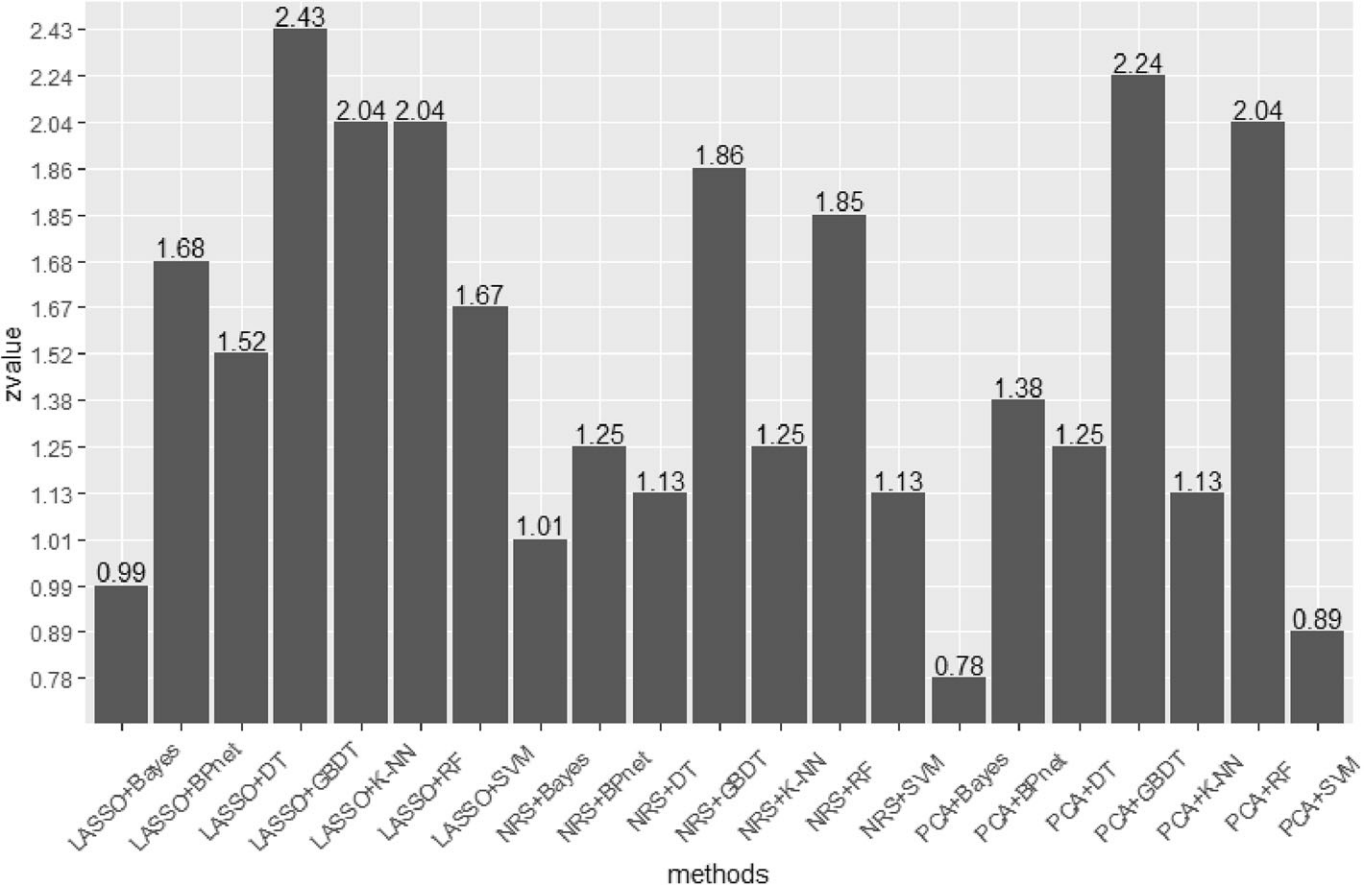
Z-values for the AUC after Z-tests. LASSO+GBDT, LASSO+K-NN, LASSO+RF, PCA+DT, and PCA+RF have Z-values greater than 1.96 (*p*<0.05). The results show that those five methods are superior to the others and that LASSO+GBDT performed best

**Fig. 8:**
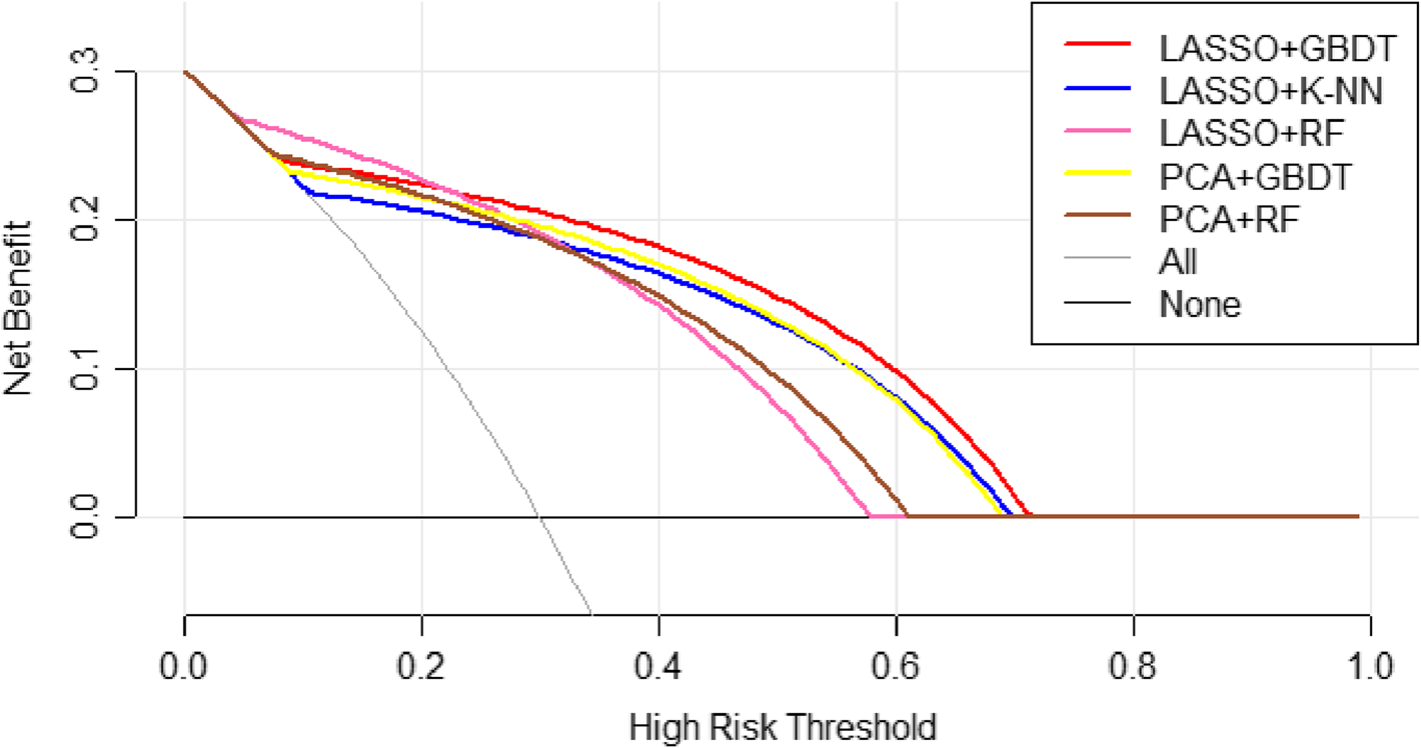
Decision Curve Analysis for LASSO+GBDT, LASSO+K-NN, LASSO+RF, PCA+DT, and PCA+RF. The X-axis represents the threshold probability and the Y-axis represents the net benefit. The LASSO+GBDT method is better than other methods when the threshold probability is greater than 0.22. Under those conditions, the model established by LASSO+GBDT will be more effective in diagnosing MVI

Figure 7 shows the Z-value of AUCs after the Z-tests. When the Z-value exceeded 1.96, the *p*-value was less than 0.05. We further compared the DCA results of these five methods, as shown in Fig. 8.

## Discussion

This study aimed to explore the feasibility of applying radiomics for preoperative diagnosis of MVI in HCC patients and to compare the differences between various dimensionality reduction and modeling methods to obtain the best diagnostic model for preoperatively diagnosing MVI. We found that although radiomics can diagnose MVI in HCC patients before surgery, different radiomics methods will result in final models with different diagnostic performance, leading to differences in MVI diagnoses. Therefore, maximizing the diagnostic performance of the model is an important way to enable accurate recognition of MVI before surgery, which is very important for HCC patients. Although the diagnostic performance of the diagnostic model only slightly improved, it increased the likelihood of identifying MVI in patients before surgery, which directly determines the future development and prognosis of the disease.

With the continuous development of radiomics, radiomics has been gradually applied in the preoperative diagnosis or evaluation of the curative effects of various tumors [3, 20–22]. Previous studies have shown that radiomics can be used in noninvasive diagnosis of preoperative MVI [17, 18]. This study can also confirm this finding, and radiomics is expected to be a new method for preoperative diagnosis of MVI. Xu et al. established a radiomics model to predict MVI in HCC by combining clinical features with radiological images [17], and the researchers finally obtained a model with an AUC of 0.889. Jie Peng et al. established a radiomics nomogram to predict MVI through LASSO+Logistic regression, and the final AUC was 0.844 [19]. However, previous studies mostly used a single method for their radiomics research, and the dimension reduction and modeling methods used in different studies were different. Although the final models obtained by these studies had high diagnostic performance, the results only showed that radiomics can be applied for the diagnosis of MVI, since these models did not achieve maximal diagnostic performance; because of the diversity in the research methods, it is difficult to achieve standardization and unification [23], which is one of the great problems radiomics is currently facing [24]. It is very difficult to popularize radiomics for disease diagnosis on a large scale before the standardization problem is solved.

In our study, by comparing 21 modeling methods commonly used in radiomics, we found that the LASSO+GBDT method had the highest accuracy, the LASSO+RF method had the highest sensitivity, and the LASSO+BPNet method had the highest specificity. These three methods used LASSO for dimensionality reduction. We speculated that the degree of over-fitting and data redundancy from LASSO after dimension reduction is lower, but this speculation needs further research. At present, some studies have used LASSO to reduce dimensions [25, 26]. Dimension reduction refers to projecting the original data from high-dimensional space to low-dimensional space through linear or nonlinear mapping and finding as many hidden rules within the high-dimensional data as possible to extract features for classification or recognition; dimension reduction is one of the important components of radiomics. The different methods for dimension reduction directly influence the effects of the final model.

Modeling refers to creating a simplified description of the object of study to achieve a certain purpose. The model simply expresses a complex research result, which is convenient for practical work. By combining different dimension reduction methods and modeling methods and comparing the AUCs of different models, we found that the LASSO+GBDT method had the highest AUC of all radiomics methods and had the highest accuracy. The sensitivity and specificity of the model were relatively high. Through further statistical analysis of the AUC, it can be seen in Fig. 7 that LASSO+GBDT, LASSO+K-NN, LASSO+RF, PCA + DT, and PCA + RF had Z-values greater than 1.96 (*p*<0.05). Among these methods, LASSO+GBDT had the highest Z-value, which indicated that the model built by LASSO+GBDT was better than other models. Then, we compared the DCA results of these five methods. It can be seen from Fig. 8 that the LASSO+GBDT method was better than other methods when the threshold probability was greater than 0.22. In this situation, the model established by LASSO+GBDT was more effective for distinguishing patients with MVI. Although the threshold probability of the LASSO+GBDT method was not always superior to others, this method was still the most effective over a considerable threshold probability range. Thus, it was concluded that LASSO+GBDT method could obtain more satisfactory results than the remaining 20 methods for diagnosing MVI before surgery. Furthermore, we speculated that the LASSO + GBDT method may also provide satisfactory predictive or diagnostic radiomics models to study other diseases, but more studies are needed to confirm this hypothesis.

There were still some limitations in our study. First, this study is a retrospective analysis, and pathological findings cannot be compared with radiological images. Second, the imaging data obtained in this study did not originate from the same CT device, and differences between images obtained by different devices were inevitable; these differences may have had an impact on the final results. Third, the study only used image data for modeling and did not combine modeling with laboratory tests and clinical parameters. Finally, this study only analyzed several of the most commonly applied dimensionality reduction and modeling methods and did not comprehensively study all current dimensionality reduction and modeling methods.

## Conclusion

In conclusion, we established several models for preoperative diagnosis of MVI in HCC patients and compared the differences and diagnostic performance of the different models, which gives the reader a choice of preferred method to use in practice. Finally, we obtained the best radiomic model for diagnosing MVI (LASSO+GBDT). This study not only indicates the feasibility of applying radiomics for preoperative noninvasive diagnosis of MVI but also tries to determine the best model-building methods and systematic research methods in radiomics research, which provides some basis for the standardization of radiomics. In the future, we need more relevant research to further explore the standardization of radiomics so that the results of radiomics studies can be applied in clinical practice as soon as possible.

## Data Availability

The datasets generated and/or analyzed for the current study are available at the Affiliated Hospital of QingDao University.
